# Cinnamon as Dietary Supplement Caused Hyperlipidemia in Healthy Rats

**DOI:** 10.1155/2021/9892088

**Published:** 2021-07-02

**Authors:** Xiaomin Huang, Haiyang Cai, Han Li, Yixun Su, Hui Li, Weihong Li, Chenju Yi, Brian G. Oliver, Hui Chen

**Affiliations:** ^1^Sun Yat-Sen University, The Seventh Affiliated Hospital of Sun Yat-Sen University, Shenzhen, China; ^2^Faculty of Basic Medical Sciences, Chengdu University of Traditional Chinese Medicine, Chengdu, Sichuan, China; ^3^School of Life Sciences, University of Technology Sydney, Sydney, New South Wales, Australia

## Abstract

**Objective:**

Cinnamon is a cooking spice and a medicinal herb. It is increasingly used as a health supplement due to its perceived benefit to prevent and or manage type 2 diabetes and metabolic disorders. However, it is unclear if regular consumption of this medicinal plant will interfere with normal physiological functions. Therefore, this study investigated the impact of daily cinnamon supplements on glucose and lipid metabolic profiles in healthy rats.

**Methods:**

Male rats (Sprague Dawley, 8 weeks) were supplied with cinnamon in their diet (equivalent to ∼1 g/day in humans) for two weeks. Blood glucose and lipid levels, as well as metabolic markers in both liver and abdominal white adipose tissue, were measured.

**Results:**

Cinnamon significantly increased fat mass and blood cholesterol and low-density lipoprotein (LDL) levels, but reduced fasting blood glucose level by 12%. Liver functional enzymes were normal in rats consuming cinnamon. However, several lipid metabolic markers were impaired which may contribute to dyslipidemia, including two main switches for energy metabolism (sirtuin 1 and peroxisome proliferator-activated receptor-gamma coactivator-1*α*) and the LDL receptor. However, *de novo* lipid synthesis enzymes and inflammatory markers were also reduced in the liver by cinnamon treatment, which may potentially prevent the development of steatosis. Markers for lipid oxidation were downregulated in fat tissue in cinnamon-treated rats, contributing to increased fat accumulation.

**Conclusion:**

Daily low-dose cinnamon supplementation seems to promote abdominal adipose tissue accumulation and disturb lipid homeostasis in healthy rats, raising the concerns regarding daily use in healthy people.

## 1. Introduction

Cinnamon is a commonly used food spice in many countries. As a medicinal herb, cinnamon has also been traditionally used to promote coronary and microcirculation in the extremities, as well as in treating diabetes in several countries [1–3]. Human trials have provided evidence supporting the blood glucose lowing effect of cinnamon in patients with type 2 diabetes [4, 5]. This effect seems largely attributed to cinnamaldehyde [6]. As such, commercial cinnamon supplements (1-2 g per day) also claim beneficial effects on blood glucose control and cardiovascular health. Due to its perceived health benefits, cinnamon is increasingly used as a supplement, especially among individuals with diabetes [7].

While the antidiabetic effect of cinnamon is supported by evidence from clinical trials, its impacts on blood lipids vary between studies. In humans and animals with diabetes, cinnamon supplements reduced blood low-density lipoprotein (LDL) and cholesterol levels, whereas a recent systematic review and meta-analysis on human trials of cinnamon showed no effect on blood LDL levels [5, 8–11]. The confirmed efficacy of cinnamon powder on blood lipid control has been shown mainly in patients with type 2 diabetes, which can be affected by confounding factors, such as strategies to promote healthy lifestyles that reduce both blood glucose and lipids and the introduction of other medications to manage complications (e.g., antihypertensive drugs). It is also possible that cinnamon alone may not be potent enough to counteract complex risk factors (e.g., drinking and social eating). However, in well-controlled animal studies, lipid-lowering effects of cinnamon powder and cinnamon extracts have been shown in rats fed a high-fat diet [12–14], although the chemical extracts have not been approved to be used by humans.

Cinnamon is also consumed by healthy people in the form of powder or dry bark. Due to the perceived benefit in people with metabolic disorders, there is increasing popularity for it to be used as a health supplement to prevent the development of such disorders. As a medicinal plant, it is likely that there will be both positive and negative effects of some of the active chemical species. While identifying individual chemicals is of interest to medicinal chemists, the raw unextracted plant is the likely form to be consumed by the general public. Blood glucose and lipids are commonly used biomarkers for the initial screening of metabolic disorders in the clinic. Therefore, it is useful to investigate the influence of unextracted cinnamon on blood glucose and lipid profiles in the absence of disease. This formed our first aim, which was to investigate how cinnamon bark affects the blood lipid and glucose profile in healthy rats fed a standard diet (i.e., rodent chow balanced with nutrients).

The liver plays a key role in lipid metabolism, especially in the synthesis of cholesterol and major lipoproteins (e.g., LDL and HDL). Suppressing the rate-controlling enzyme in cholesterol synthesis, 3-hydroxy-3-methyl-glutaryl-coenzyme A reductase (HMGCR), in the liver, can induce the expression of LDL receptors (LDLR). This further increases the breakdown of LDL and reduces blood cholesterol levels. Squalene monooxygenase (SQLE) is another rate-limiting enzyme in sterol biosynthesis. Also, sirtuins (SIRTs) are a group of deacetylases and mono-ADP-ribosyl transferases that function as the main switch for energy metabolism [15]. SIRT1-peroxisome proliferator-activated receptor-gamma coactivator-1*α* (PGC-1*α*) pathway plays a vital role in cellular substrate metabolism [16]. Therefore, all the abovementioned metabolic markers were measured in the liver and adipose tissue, which are vital mechanisms for systemic glucose and lipid metabolic homeostasis.

## 2. Methods

### 2.1. Animal Study

The study was approved by the Animal Ethics Committee of the Chengdu Dossy Experimental Animal Co., Ltd. (SCXK (Chuan) 2015-030). Sprague Dawley rats (male, 8 weeks, *n* = 10) were fed either standard rodent chow (14% fat) or rodent chow containing cinnamon powder (1 g/kg chow) for two weeks. Medical grade cinnamon (Sichuan New Lotus Chinese Medicine Pieces Co., Ltd.) for human use was added to the standard rodent chow by Chengdu Dossy Experimental Animal Co., Ltd. The average daily food consumption of the rats was 25.5 g/day/rat as measured by our laboratory in this strain, making the treatment dose equivalent to ∼1 g/day in humans of 75 kg based on the calculation using a published method [17]. At the endpoint, after overnight fasting and anesthesia (pentobarbital sodium, 40 mg/kg), blood was collected for serum via cardiac puncture, and blood glucose was measured using a glucose meter (Accu-Chek®, Roche Diagnostics). The organs were dissected and weighed. White adipose tissue and liver were snap-frozen and kept at −80°C.

### 2.2. Bioassays

Commercial ELISA kits were used to measure serum lipids LDL (Cat. F4562, Shanghai Westang Biotechnology) and cholesterols (Cat. ab65390, Abcam, Cambridge, United Kingdom), according to the instructions provided by the manufactures. The liver enzymes alanine aminotransferase (ALT) and aspartate transaminase (AST) in the serum were also measured using commercial kits (ALT kit Cat. C009-3-1 and AST kit Cat. C010-3-1, Nanjing Jiancheng Bioengineering Institute, China), following the manufacturer's instructions.

### 2.3. Real-Time PCR

mRNA expression of energy metabolic markers was measured in the liver and white adipose tissue using real-time PCR. The tissues were homogenized in RNAzol (Sigma-Aldrich, USA) and total RNA was isolated according to the manufacturer's instructions [18, 19]. Quantiﬁcation was performed with a two-step reaction process: reverse transcription and qPCR. Reverse transcription was carried out with the M-MLV reverse transcriptase kit (Accurate Biology, China) according to the manufacturer's protocol in a GeneAmp® PCR System 9700 (Applied Biosystems 7500 Real-Time PCR System, USA). qPCR experiments were carried out using the SYBR Green qPCR kit from KAPA on an Applied Biosystems 7500 Real-Time PCR System. The primer sequences were designed in the laboratory and synthesized by TSINGKE Biological Technology (Guangzhou, China) based on the mRNA sequences obtained from the NCBI database (Table 1). At the end of the PCR cycles, the melting curve analysis was performed to validate the speciﬁc generation of the expected PCR product. mRNA expression was calculated using 2^−∆∆Ct^ methods using the 18s as the housekeeping gene [18, 19]. The control group was assigned as the calibrator against which all other results were expressed as fold changes [18, 19].

### 2.4. Statistical Methods

The results are expressed as mean ± SEM. The data were analyzed by unpaired student's t-test. *P* < 0.05 was considered statistically significant.

## 3. Results

Cinnamon supplementation did not affect body or organ weights (Table 2). However, it increased retroperitoneal white fat mass (*P* < 0.05, Table 2). It also reduced fasting blood glucose levels by 12%, consistent with the literature [20–22]. However, cinnamon supplementation increased blood LDL (*P* < 0.01 vs. control, Table 2) and cholesterol levels (*P* < 0.05 vs. control, Table 2).

**Figure 1:**
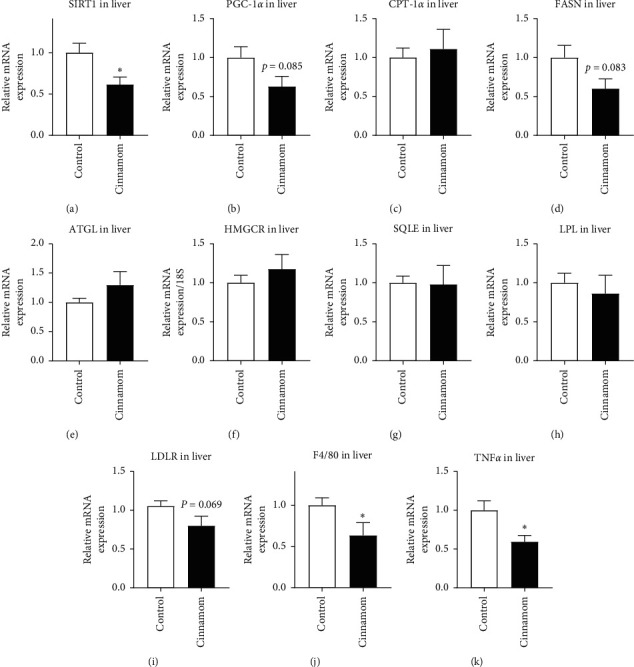
mRNA expression of metabolic markers, SIRT1 (a), PGC-1*α* (b), CPT-1*α* (c), FASN (d), ATGL (e), HMGCR (f), SQLE (g), LPL (h), LDLR (i), F4/80 (j), and TNF*α* (k), in the liver. Results are expressed as mean ± SE, *n* = 8–10. ^*∗*^*P* < 0.05.

In the liver, mRNA expression of several lipid metabolic markers including SIRT1 (*P* < 0.05, Figure 1(a)), PCG-1*α* (*P*=0.085, Figure 1(b)), fatty acid synthase (FASN, *P*=0.083, Figure 1(d)), and LDLR (*P*=0.069, Figure 1(i)) was reduced by cinnamon supplementation. Also, the markers for macrophage number F4/80 (Figure 1(j)) and the proinflammatory cytokine TNF*α* (Figure 1(k)) were downregulated by cinnamon supplementation (*P* < 0.05 for both).

**Figure 2:**
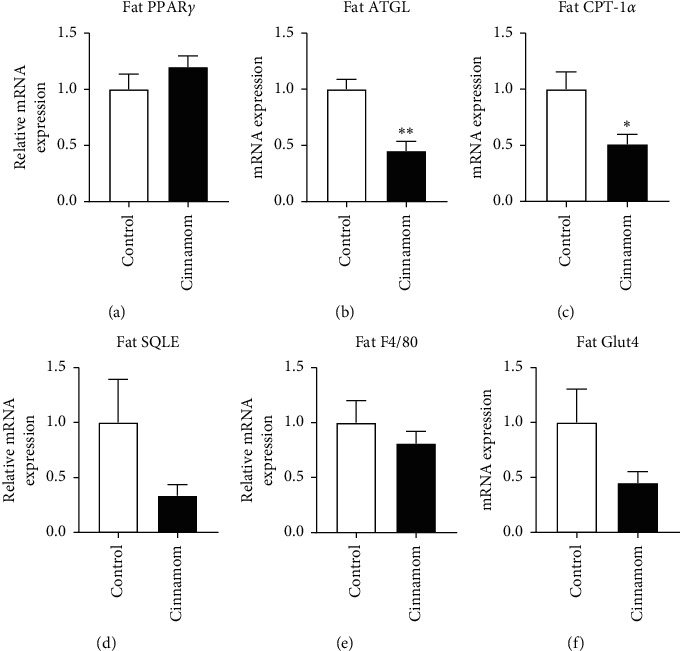
mRNA expression of metabolic markers, fat PPAR*γ* (a), ATGL (b), CPT-1*α* (c), SQLE (d), F4/80 (e), and Glut4 (f), in the abdominal white adipose tissue. Results are expressed as mean ± SE, *n* = 6. ^*∗*^*P* < 0.05; ^*∗∗*^*P* < 0.01.

In the fat tissue, markers for lipolysis, adipose triglyceride lipase (ATGL, *P* < 0.05, Figure 2(b)), and free fatty acid oxidation, CPT-1*α* (*P* < 0.05, Figure 2(c)), were significantly reduced, with halved levels of SQLE (Figure 2(d)) and glucose transporter 4 (Glut4, Figure 2(f)), albeit without statistical significance.

## 4. Discussion

In the literature, the impact of cinnamon supplements on metabolic disorders has only been examined in the context of diabetes or high-fat diet consumption. The outcomes promoted the use of cinnamon supplements even among healthy individuals, although without any evidence of the health benefit. This makes the major findings of this study particularly important to inform the use in healthy people, in that low-dose cinnamon dietary supplementation may increase blood cholesterol and LDL levels, as well as abdominal fat mass. We also found that such adverse impacts on lipid metabolic profiles may be related to impaired lipid metabolic markers in both the liver and fat tissue.

Liver function is critical for maintaining systemic glucose and lipid metabolic homeostasis [23]. Blood ALT and AST levels are used in the clinic as an indicator of hepatocyte integrity, which are highly concentrated in healthy hepatocytes with a low amount released into the circulation. The blood levels of both enzymes rise when hepatocytes are damaged during conditions such as drug-induced liver damage and liver steatosis. In this study, unchanged blood ALT and AST levels suggest that daily consumption of a low dose of cinnamon is safe for hepatocyte integrity. However, it is not so optimistic for the circulating lipid profile in rats consuming cinnamon daily, reflected by increased cholesterol and LDL levels in the blood. This effect is opposite to the beneficial effects observed in those with preexisting metabolic conditions. It is well accepted that high cholesterol levels in the blood are strongly associated with vascular disorders. Cholesterol in the circulation is mainly synthesized in the liver. LDL is rich in cholesterol, and when increased, it is associated with the development of atherosclerosis and cardiovascular and cerebrovascular diseases [24]. In the literature, cinnamon has been shown to reduce blood lipid levels in patients with type 2 diabetes [10]. Such effects may be secondary to the improvement in glycaemic control, where ketoacidosis, a common complication due to reduced cellular glucose uptake, can result in hyperlipidemia due to the mobilization of alternative fuels (i.e., lipids restored in the fat tissue). In the current study, in healthy rats, both LDL and cholesterol were increased by cinnamon supplement, albeit with normoglycemia. This may attribute to several downregulated lipid metabolic markers in the liver. SIRT1 is essential for cellular survival, especially during stress, which promotes nutrient metabolism to maximize energy availability [16]. SIRT1 activates its downstream signal PGC-1*α*, which is another essential metabolic switch to promote fatty acid oxidation [25]. Thus, the suppression of SIRT1 by obesity leads to metabolic dysfunction, such as hyperlipidemia and hyperglycemia [26]. In this study, liver SIRT1 and PCG-1*α* are downregulated in cinnamon-treated rats, which may indirectly lead to their dyslipidemia. Several enzymes and mediators involved in cholesterol and LDL production in the liver were not significantly affected by cinnamon, perhaps due to the short duration of the treatment. However, a trend of increased HMGCR and reduced LDLR can be observed, which may contribute to the increased blood total cholesterol and LDL levels.

However, it needs to be noted that liver FASN, the enzyme that regulates *de novo* lipid synthesis, was suppressed by cinnamon supplementation. Concurrently, there was also a reduction in macrophage (Kupfer cells) number and inflammation, reflected by F4/80 and TNF*α* expression, respectively. Both increased FASN and Kupfer cells have been shown to promote the development of liver steatosis, while the suppression of FASN and Kupfer cells can ameliorate alcohol-induced steatosis [27–29]. These changes in our study suggest that cinnamon may help to prevent liver steatosis. This effect may be promising in controlling the fatty liver disease. In a small randomized clinical trial on patients with nonalcoholic fatty liver disease (NAFLD), 1.5 g of cinnamon was administered daily for 12 weeks with the implementation of both a balanced diet and physical activity [30]. Although the study reported improved blood lipid profile and reduced blood ALT, AST, and inflammatory markers suggesting improved liver function, it did not directly examine the liver lipid accumulation using imagining analysis [30]. While the therapeutic effect of cinnamon is important to those with lipid metabolic disorders, the preventive effects of cinnamon against NAFLD may be of more interest to individuals either at risk or no risk (i.e., healthy population), which needs to be confirmed in models of hepatic steatosis in future studies.

The increase in abdominal fat mass by cinnamon is small, but yet is of concern. Abdominal fat is now considered a risk for early all-cause mortality, with the highest incidence in cardiovascular diseases and metabolic disorders [31]. In this study, fat mass was increased in the face of hyperlipidemia in cinnamon-treated rats. This fat accumulation does not seem to be due to fat cell differentiation reflected by unchanged PPAR*γ*, which regulates the differentiation of new adipocytes. The reduced breakdown and oxidation of the lipids in the fat tissue may be the contributors, as suggested by the lipolysis marker ATGL and fatty acid oxidation markers CPT-1*α* and SQLE. When fat storage is increased, blood monocytes are attracted to the fat tissue forming resident macrophages, which in turn produce the proinflammatory cytokine TNF*α* leading to insulin resistance and glucose intolerance. However, in this study, the initial fat accumulation has not increased macrophage number or inflammation. This may be due to the short duration of treatment and the small increase in fat mass from 1.13% to 1.50% of total body weight. Long-term treatment (e.g., 12 weeks) is needed in future studies to confirm this observation further.

It also needs to be noted that Glut4 is downregulated in the fat. Glut4 is essential for insulin-stimulated glucose uptake [32]. Cinnamon has been suggested to improve insulin resistance caused by high-fat diet consumption [32, 33]. However, in the setting of a balanced healthy diet, the response of Glut4 observed in this study may suggest otherwise. Also, cinnamaldehyde in cinnamon has been identified to exert the hypoglycemia effect [34]. In this study, we used cinnamon in rats with normoglycemia, which may act differently. It is likely that the downregulation of Glu4 only occurs during normoglycemia to prevent hypoglycemia, and cinnamaldehyde increases Glu4 level when there is increased blood glucose or the presence of insulin resistance [6, 35].

There are limitations to this study. We only tested the metabolic effect of a single low dose of cinnamon in a short-term setting, which showed significant dyslipidemia and increased fat mass in healthy rats. A 16-week cinnamon treatment using a higher dose (3 g/day) has been shown to improve body composition and metabolic parameters in an Indian cohort with preexisting metabolic disorders [36]. Therefore, future studies need to evaluate the effects of high doses to determine whether such metabolic benefits of cinnamon only occur in the setting of metabolic disorder.

## 5. Conclusion

A low level of cinnamon supplementation in the daily diet seems to encourage fat accumulation and disturb lipid homeostasis in healthy rats, opposite to the beneficial effects reported in individuals with metabolic disorders. Thus, healthy individuals or those with liver dysfunction may need to be cautious with the frequent consumption of cinnamon.

## Figures and Tables

**Table 1:** Primers used for real-time PCR.

Gene symbol	Forward primer (5 ≥ 3)	Reverse primer (5 ≥ 3)
ATGL	CGCAATCTCTACCGCCTCTC	GGGTTGGTTCAGTAGGCCATT
CPT-1*α*	CGGCAGACCTATTTTGCACG	TGGACTTGTCAAACCACCTGT
FASN	AGCCTGAGCTTGTCCCTAGA	CACTGGTACACTTTCCCGCT
F4/80	CCACAACACCTACCTGCACC	ATGATAGCGCAAGCTGTCTGG
Glut4	TTCCAGTATGTTGCGGATGCT	AATGTCCGGCCTCTGGTTTC
HMGCR	TGCAGAGCGATCAGTCTTGG	AATCTGCTCGTGCTGTCGAA
LPL	GGTCGCCTGGTCGAAGTATT	CAGCTGGTCCACATCTCCAA
LDLR	AGACCCAGAGCCATCGTAGT	GGCCACTGGGAAGATCTAGTG
PGC-1*α*	TGGAGTGACATAGAGTGTGCTG	TATGTTCGCGGGCTCATTGT
SIRT1	TTTATGCTCGCCTTGCTGTG	GCTTCAATGCTGTTTCTTCTTTGC
SQLE	TCAGTGAACAAACGAGGCGT	GCCTGGAAAATAGCGGCATC
TNF*α*	ATGGGCTCCCTCTCATCAGT	GCTTGGTGGTTTGCTACGAC
18S	ATTCCCAGTAAGTGCGGGTC	AAGTTCGACCGTCTTCTCAGG

**Table 2:** The effect of the cinnamon supplement on anthropometry markers.

	Control	Cinnamon
Body weight (g)	460 ± 14	468 ± 9
Liver (g)	13.2 ± 0.7	13.2 ± 0.3
Liver (%)	2.88 ± 0.08	2.82 ± 0.03
Kidney (g)	2.59 ± 0.11	2.53 ± 0.05
Kidney (%)	0.56 ± 0.02	0.54 ± 0.01
White adipose tissue (g)	5.18 ± 0.41	7.10 ± 0.79^*∗*^
White adipose tissue (%)	1.13 ± 0.08	1.50 ± 0.15^*∗*^
Skeletal muscle (g)	0.87 ± 0.03	0.88 ± 0.04
Skeletal muscle (%)	0.19 ± 0.01	0.19 ± 0.01
Fasting blood glucose (mM)	9.38 ± 0.26	8.35 ± 0.50
Cholesterol (mM)	2.16 ± 0.08	2.36 ± 0.06^*∗*^
LDL (mM)	0.94 ± 0.10	1.36 ± 0.07^*∗∗*^
ALT (U/L)	24.1 ± 1.34	25.7 ± 2.51
AST (U/L)	53.8 ± 4.73	53.0 ± 5.66

Results are expressed as mean ± SE, *n* = 10. ^*∗*^*P* < 0.05; ^*∗∗*^*P* < 0.01. ALT, alanine aminotransferase; AST, aspartate transaminase; LDL, low-density lipoprotein.

## Data Availability

All data are included in this manuscript.
